# Insulin Edema With Use of U-500 Regular Insulin in a Hybrid Closed-Loop Insulin Pump

**DOI:** 10.7759/cureus.10886

**Published:** 2020-10-10

**Authors:** Mostafa Vasigh, Janan Mohammady, Rachel Hopkins

**Affiliations:** 1 Internal Medicine, State University of New York Upstate Medical University, Syracuse, USA; 2 Internal Medicine, Tehran University of Medical Sciences, Tehran, IRN; 3 Endocrinology, Diabetes and Metabolism, State University of New York Upstate Medical University, Syracuse, USA

**Keywords:** insulin edema, diabetes mellitus, insulin, insulin pump, edema

## Abstract

Insulin edema is a rare complication of insulin therapy which has been described in known or newly diagnosed people with diabetes, following initiation or intensification of insulin treatment. Here we present a 63-year-old man with complaints of weight gain, shortness of breath, and lower extremity edema starting two weeks after the change of his insulin pump to the hybrid closed-loop insulin pump system and substitution of U-100 aspart insulin with U-500 regular insulin. Laboratory studies, imaging, and electrocardiogram (EKG) were performed to evaluate the cause of acute edema and were all normal. Hemoglobin A1C showed remarkable improvement after the pump change and the insulin pump download showed a significant increase in the amount of total daily insulin administered. With the exclusion of other causes of acute edema, the patient was diagnosed with insulin edema. He was started on spironolactone 50 mg/daily and showed a desirable improvement of edema on follow-up.

This case shows that although the use of the hybrid insulin-pump system helps to obtain better control of diabetes in many patients, the rapid improvement in glycemic control may precipitate the development of insulin edema. Furthermore, the use of high concentration insulin in insulin pumps is off-label and their use might increase the rate of complications of insulin therapy including insulin edema.

## Introduction

While its true incidence is unknown, insulin edema is considered a rare complication of insulin therapy. It has been described following the initiation or intensification of insulin treatment in known or newly diagnosed people with diabetes [1,2]. This complication presents with localized lower extremity edema or, less frequently, generalized edema [1,3]. Being a diagnosis of exclusion, insulin edema may be underreported, and due to its rare incidence it is generally not included in the differential diagnosis of edema, which can result in delayed diagnosis and treatment.

Here, we report a case of insulin edema in a patient who was started on the Medtronic MiniMed 670G (Medtronic, Minneapolis, MN, USA) hybrid closed-loop artificial pancreas system, with the development of edema following substitution of U-100 aspart insulin with U-500 regular insulin. The hybrid closed-loop (Auto Mode) feature was used to better manage his poorly controlled diabetes.

## Case presentation

A 63-year-old male with type 2 diabetes mellitus of 30 years duration, complicated by obstructive sleep apnea, hyperlipidemia, bilateral proliferative retinopathy, obesity, and prostate cancer presented to the emergency department with complaints of shortness of breath, weight gain of 20 lbs, and increased abdominal girth occurring over the course of two weeks.

The patient has a history of poorly controlled diabetes mellitus with hemoglobin A1c (HbA1c) greater than 9% with the use of insulin pump therapy for over 10 years. He suffers from bilateral diabetic retinopathy which is being followed by an ophthalmologist but does not have any history of peripheral neuropathy, angina, intermittent claudication, or transient ischemic attacks (TIAs). He also does not have any history of diabetic ketoacidosis. 

He had visited his endocrinologist five months prior to the current presentation for a routine follow-up visit. He was using the Medtronic MiniMed 530G pump with U-200 lispro insulin with continuous glucose monitoring. Shortly after that visit, his insulin pump was upgraded to the Medtronic MiniMed 670G pump with Guardian 3 sensor without any changes in the pump settings or insulin formulation. The patient was instructed in the use of the “Auto Mode” feature and began using that feature more than 70% of the time.

About 10 weeks later, due to formulary issues, the insulin type was changed to U-100 aspart and the pump settings were adjusted to reflect the change in concentration. After three weeks, the patient had another follow-up visit at which point his HbA1c was 8.3% showing remarkable improvement. He did not have any complaints of edema or shortness of breath at that time and his weight was at his baseline. He did complain of the need for frequent pump reservoir refills due to the high doses of daily insulin requirements and asked that his insulin be changed to a more concentrated formula. After a discussion of the potential risks of doing so and at his request he was switched to U-500 regular insulin and pump adjustments were again made accordingly.

About two weeks after the initiation of U-500 regular insulin, the patient started noticing an increase in his weight and the development of bilateral lower extremity edema. Within two weeks increase in abdominal girth and shortness of breath ensued for which he eventually presented to the emergency department.

Laboratory studies including comprehensive metabolic panel (albumin: 4.3 g/dl), troponin, pro-B-type natriuretic peptide (proBNP), thyroid stimulating hormone (TSH), and urinalysis as well as electrocardiogram were all unremarkable. He had mild microalbuminuria with urine microalbumin 32.2 mg/L and urine microalbumin/creatinine 18.7 µg/mg creatinine. Imaging studies including chest X-ray, CT angiography (CTA) scan thorax, and CT scan abdomen/pelvis did not show any pulmonary embolism, pleural effusion, or free pelvic fluid. Echocardiogram was done and did not reveal any findings suggestive of systolic or diastolic heart failure. The patient was discharged from the ED and advised to follow up with his primary care provider (PCP).

During the follow-up visit with his PCP, he was diagnosed with insulin edema based on the exclusion of other causes of acute generalized edema. He was started on a 10-day course of spironolactone 50 mg/daily, advised on salt and fluid restriction, and asked to monitor his weight daily. He was also referred to his ophthalmologist for evaluation of the development of macular edema.

A follow-up visit on week 21 post-pump change showed a decrease of 10 lbs in body weight with significant improvement in the edema and shortness of breath. The ophthalmology visit had ruled out macular edema (Table 1).

**Table 1:** Bodyweight, hemoglobin A1c, insulin concentration, and average total daily insulin administration * 14 weeks prior to upgrading the insulin pump from MiniMed 530G to MiniMed 670G

	Insulin Type	Hemoglobin A1c (%)	Body Weight (Kg)	Average Total Daily Insulin Since Last Visit (units/day)	Percent Low (%)	Percent Auto Mode (%)	Time in Target Range (%)
Week -14*	Lispro U-200	9.4	113	154			
Initiation of 670G pump	Lispro U-200	9.3	117	167			
Week 10	Aspart U-100	—	117	224			
Week 13	Aspart U-100	8.3	118	253	0	75	32
Week 15	Regular U-500	—	118	252			
Week 19 (ED visit)	Regular U-500	—	124	926			
Week 21	Regular U-500	8.3	119	515	2	79	54

## Discussion

Although the incidence of insulin edema was reported to be as high as 3.5% in a small group of patients with diabetes on insulin therapy from Kampala, the incidence in the United States is unknown [4]. Several clinical features have been previously described as risk factors for developing insulin edema. These include age of 20-40 years, type 1 diabetes, poor glycemic control, low body weight, poor nutritional status, new-onset diabetes, and higher doses of insulin therapy [1,5].

Several theories have been postulated regarding the pathophysiological mechanisms of insulin edema. These include the direct antinatriuretic effect of insulin on renal tubules, increased glucocorticoid retention due to insulin-induced hypoglycemia, and increased vascular permeability [1]. The first two mechanisms are more likely explanations for this phenomenon in our patient since there was no evidence of hypoalbuminemia or third-space fluid accumulation in the thorax and abdomen. The rapid weight gain seems to have been due in part to fluid retention and, as in most cases, edema was responsive to diuretic therapy. However, part of the weight gain might also be due to increased glucose uptake and adiposity with improved glycemic control.

In previous case reports, patients with insulin-induced edema were substantially malnourished or underweight [3,5,6]. Our patient, who had longstanding diabetes, was not insulin naive and had excessive body weight prior to the development of edema. This is similar to a previously reported case of a patient who was not insulin naive and developed insulin edema a few days after commencing insulin-pump therapy, which resulted in a dramatic and abrupt improvement of glycemic control [7]. Our patient experienced a more gradual decrease in HbA1c and only started developing edema once his insulin type was changed to U-500 regular insulin without a clear subsequent decrease in HbA1c. Although he did not develop any severe hypoglycemia, he did have an increased occurrence of blood sugars in the mild hypoglycemia range. In other words, although HbA1c remained approximately the same he did have more time with lower blood sugars which might have contributed to the development of edema.

The occurrence of edema in our patient within two weeks of using U-500 regular insulin implicates the complexities of converting from U-100 to more concentrated insulins as described in a recent review on concentrated insulins in current clinical practice. Although several studies have demonstrated that the use of U-500 in traditional insulin pumps can be safe and effective [8,9], such use in commercially available insulin pumps is off-label. Algorithms used by hybrid closed-loop systems are written for U-100 insulins and specifically for the pharmacokinetics of rapid-acting insulin analogs, not regular insulin or U-500 regular insulin, which have more complicated pharmacokinetics.

Moreover, altered insulin sensitivity as well as insulin concentration can change the pharmacodynamic characteristics of insulin [10]. Higher insulin concentrations are associated with slower diffusion into tissues, resulting in a more prolonged insulin absorption time and effect [10,11]. These characteristics raise the possibility of altered insulin levels in patients using equivalent doses of a higher concentration of insulin. Further studies are required to investigate whether insulin-induced complications such as insulin edema are more likely to occur with the use of concentrated insulins.

Another important point to consider is that our patient was primarily using the hybrid closed loop system of the Medtronic MiniMed 670G pump (around 70% of the time). This resulted in both improved glycemic control and an increase in daily insulin delivery. The use of hybrid closed-loop systems has been found to result in significant improvement in “time in glucose target range” [12], as was the case in this patient.

## Conclusions

Insulin edema is a potential side effect of commencing or intensifying insulin therapy in people with diabetes. Given that rapid improvement in glycemic control is a risk factor for both insulin edema and worsening of diabetic retinopathy, perhaps initiation of hybrid closed-loop therapy should be taken in a stepwise fashion with higher initial glycemic targets set for patients starting with a very high hemoglobin A1c. The following questions, however, remain to be answered regarding this rare clinical condition: 1) Is there a direct relationship between the concentration of insulin used in an insulin pump and the development of insulin edema? 2) Does the "Auto Mode" setting of the insulin pump increase the risk of developing insulin edema?
